# Expanded polling booth surveys monitor HIV programme outcomes among female sex workers and men who have sex with men in Nairobi, Kenya

**DOI:** 10.3389/fpubh.2026.1969489

**Published:** 2026-10-27

**Authors:** Parinita Bhattacharjee, Shajy Isac, Joshua Kimani, Faran Emmanuel, Rhoda Kabuti, Antony Kinyua, Virginia Njeri Ndukuyu, Helgar Musyoki, Anthony Kiplagat, Peter Arimi, Souradet Y. Shaw, Monica Gandhi, James Blanchard, Marissa L. Becker, Leigh M. McClarty

**Affiliations:** 1 Institute for Global Public Health, University of Manitoba, Winnipeg, MB, Canada; 2 Partners for Health and Development in Africa, Nairobi, Kenya; 3 Sex Workers Outreach Project (SWOP) Clinic, Nairobi, Kenya; 4 Grant Management Division, The Global Fund, Geneva, Switzerland; 5 Ministry of Health, Nairobi, Kenya; 6 University of California, San Francisco, CA, United States

**Keywords:** female sex workers, HIV, Kenya, men who have sex with men, programme outcomes, urine tenofovir assay

## Abstract

**Introduction:**

Nairobi County has the largest HIV prevention programme for female sex workers (FSW) and men who have sex with men (MSM) in Kenya. Routine monitoring of programme outcomes is critical for tracking progress toward programme targets. Monitoring approaches need to be rapid, affordable, and sufficiently robust to capture programme outcomes while meaningfully involving communities.

**Methods:**

We conducted a cross-sectional outcome assessment among FSW and MSM (71% reporting sex work) in Nairobi County from April–July 2023 using an expanded polling booth survey (ePBS). The ePBS combined anonymous group-level polling booth survey (PBS) with an individual biobehavioural survey (BBS) to assess behavioural, structural and biological outcomes. Weighted descriptive analyses summarised programme outcomes.

**Results:**

The target sample size for the study was 769 FSW and 420 MSM. Overall, 759 FSW (response rate 98.6%) and 398 MSM (response rate 94.7%) participated in 64 PBS sessions; 758 FSW completed the BBS, while 398 MSM completed the behavioural component and 396 completed the biological component. Condom use at last sex was reported by 80.8% of FSW with paying clients and by 72.7 and 68.9% of MSM with non-regular partners and paying clients, respectively. Recent peer educator contact was reported by 68.8% of FSW and 65.8% of MSM. Healthcare-related stigma was reported by 8.0% of FSW and 7.5% of MSM, and recent forced sex by 10.7% of FSW and 6.5% of MSM. HIV prevalence was 14.0% among FSW and 18.6% among MSM. Among participants living with HIV, 85.9% of FSW and 80.4% of MSM were virally suppressed. Among those reporting current oral PrEP use, tenofovir was detected in urine among 48.3% of FSW and 52.6% of MSM.

**Conclusion:**

FSW and MSM in Nairobi remain priority populations for the HIV response. The findings suggest persistent gaps across behavioural, structural and biomedical outcomes that need to be addressed. The ePBS approach offers a practical and scalable method for generating actionable outcome data that can complement routine monitoring systems and support adaptive HIV programming. As countries face increasing pressure to optimise HIV programmes within constrained funding environments, approaches that generate timely and actionable outcome data will become increasingly important.

## Introduction

Nairobi County remains a priority geography within Kenya’s HIV response because it has large populations of female sex workers (FSW) and men who have sex with men (MSM), many of whom sell sex for money or gifts (1). Current population size estimates indicate that Nairobi has the largest estimated populations of FSW, at 39,227 (30,835–47,533), and MSM, at 9,421 (7,397–11,912) (2). Key populations, including FSW and MSM, are central to Kenya’s HIV response and are prioritised in the Kenya AIDS Integration Strategic Framework 2025–2030 (3).

The National AIDS and STI Control Programme (NASCOP) and the National Syndemic Diseases Control Council (NSDCC), under Kenya’s Ministry of Health, lead the national HIV prevention response for key populations, working closely with county governments within the context of devolved health service delivery (4). In Nairobi, the Sex Worker Outreach Programme (SWOP), implemented by Partners for Health and Development in Africa (PHDA), is one of the largest key population programmes in the country, serving approximately 40,000 FSW and 10,000 MSM (5). The programme provides a comprehensive package of services aligned with national key population guidelines, including peer outreach, HIV testing, condoms and lubricants, oral tenofovir-based pre-exposure prophylaxis (PrEP), antiretroviral therapy (ART) linkage and adherence support, sexually transmitted infections (STI) services, violence response and related referrals (6).

Routine outcome monitoring is essential for understanding whether these services are reaching the intended populations and producing the expected prevention and treatment outcomes (7, 8). Kenya’s key population programmes have used several approaches to measure programme outcomes, including integrated biological and behavioural surveys in 2011 (9) and 2024 (10), and polling booth surveys in 2013 and 2017 (11). Each approach has strengths and limitations. Biobehavioural surveys generate important biological outcomes but are costly, resource-intensive and difficult to repeat frequently (12). Polling booth surveys can rapidly collect anonymous data on sensitive behaviours and service experiences, but they do not measure biological markers (13). Routine programme data are also available, but primarily measure contacts and commodity distribution rather than prevention or treatment outcomes (14). The expanded polling booth survey (ePBS) (15) approach, which combines a core polling booth survey with an individual biobehavioural survey and laboratory testing, was developed to bridge this gap by providing a practical, timely and community-engaged method for generating outcome data that can directly inform programme decision-making and adaptation.

This paper presents findings from a 2023 outcome assessment among FSW and MSM in Nairobi County using the expanded polling booth survey (ePBS) approach. We also reflect on the value of ePBS as a method for generating evidence to inform programme decision-making.

## Methods

The ePBS is a rapid cross-sectional outcome assessment method designed to collect behavioural, structural and biological programme outcome data. It builds on the original polling booth survey (PBS) methodology, which uses anonymous group-level self-reporting to assess participants’ behaviours, programme exposure and selected programme outcomes without linking responses to individual participants. The ePBS extends this approach by incorporating a biobehavioural survey (BBS) component and qualitative enquiry to assess whether service uptake is translating into intended biological and programme outcomes and to understand why gaps in effective coverage persist (15).

For this assessment, ePBS included a core PBS module and an individual-level BBS with laboratory testing. The PBS module generated anonymous group-level estimates for sensitive behavioural, structural and biomedical indicators, while the BBS module generated individual-level behavioural and biological data, including HIV, viral load, and urine tenofovir testing. The validated urine tenofovir assay is an objective adherence metric which provides information on PrEP pill taking over the past 4–7 days (16). The broader ePBS approach also included focus group discussions (FGDs) (17, 18), but this paper reports findings only from the PBS and BBS modules. Findings from the FGDs are reported elsewhere (8).

### Participant inclusion criteria

Eligible participants were adults aged 18 years or older who self-identified as either FSW or MSM and were actively engaged in sex work or cruising within Nairobi County. FSW participants had to report exchanging sex for money or gifts with a male partner in the previous 3 months, while MSM participants had to report anal sex with another male partner during the same period. All participants were also required to be willing and able to provide written or verbal informed consent for participation in the three study components.

### Sample size

The study used probability-based sampling to obtain representative samples of FSW and MSM in Nairobi County. Sample sizes were calculated to estimate HIV prevalence with 95% confidence and ±5% precision, incorporating the expected prevalence, design effect, finite population correction, and an anticipated 15% non-response rate. HIV prevalence estimates of 26% among FSW and 12% among MSM were used, resulting in target sample sizes of 769 FSW and 420 MSM. Assuming 10–12 participants per PBS session, 64 sessions were planned for FSW and 35 for MSM.

### Sampling and data collection

Data were collected from April to May 2023 using a two-stage probability-based sampling design. In the first stage, study locations were randomly selected from a previously validated list of locations (2) where FSW solicit clients or engage in sex, and where MSM meet sexual partners or engage in sexual activity. A total of 99 locations (64 FSW and 35 MSM) were selected, with one PBS session conducted at each selected location.

In the second stage, potential participants were randomly selected from these locations on a randomly assigned day of the week. Each of the 99 locations was allocated to a specific study week and day, during which participants were enrolled. On the assigned day, community researchers approached potential participants at the location using the study’s random selection procedure and invited them to participate. Each PBS session therefore comprised 10–12 participants from the same key population group who frequented or worked in similar locations to solicit clients or meet sexual partners. This method of sampling accounted for heterogeneity across the 17 sub-counties, location typologies such as bars with and without lodging, streets and strip clubs, and different times and days of the week.

As PBS is administered in a group setting, the questionnaire is deliberately kept brief. Questions are phrased in simple and concise language to support comprehension and facilitate easy participation (15). PBS has previously been implemented within Kenya’s key population programme, where it has been found to be an acceptable method among participants from different key population groups (19).

Participants provided informed consent for each ePBS module. Participation in the PBS module was required for enrolment in the study, while participants could opt out of the BBS or FGD and remain eligible to receive the study honorarium of USD 5.

PBS sessions were conducted separately with FSW and MSM in groups of 10–12 participants randomly selected from the same location typology. Each session lasted approximately 60 min and generated group-level data on behavioural, structural and biomedical programme outcomes (11, 14, 17). The PBS tool also included a brief set of study-developed indicators to assess self-reported emotional distress. This item was included as a programme monitoring indicator following consultation with FSW and MSM communities and was not intended to function as a diagnostic or validated mental health screening tool. After each PBS session, participants received pre-test counselling and were invited to participate in the BBS module. HIV testing services (HTS) counsellors conducted finger-prick rapid HIV tests according to national guidelines (20). Clinicians administered the individual face-to-face survey and collected urine samples. Participants with reactive HIV test results were asked to consent to venous blood sample collection for viral load testing (Roche Cobas 8,800 test). Urine specimens from participants who reported current oral PrEP use in the BBS were tested for tenofovir using a validated point-of-care lateral flow assay (21). Biological outcomes were analysed according to national guidelines (22).

FSW and MSM community researchers were engaged throughout the study. They contributed to tool design, pre-testing, data collection, interpretation of findings and dissemination. Preliminary results were presented to SWOP peer educators and clinic teams using visual materials, followed by facilitated discussions to interpret gaps and identify programmatic responses. This participatory process helped situate findings within service delivery realities and supported the translation of results into programme action. Community members were engaged as partners throughout the process, contributing to data collection and interpretation of findings. Community members provided important context, highlighted issues that might not have been apparent from the data alone, and helped identify practical responses for programme improvement.

### Data management

PBS and BBS data were entered directly into tablets using SurveyCTO, a secure web-based survey platform. Automated data quality checks were built into the system to improve data quality. Biological samples were transported to the PHDA laboratory, where laboratory data were managed through a laboratory information management system and exported in CSV format. Survey and laboratory datasets from the BBS module were linked using unique identifiers and merged into a single dataset for analysis.

### Data analyses

SPSS version 28.0 was used to conduct descriptive analyses of the PBS and BBS data presented in this paper. The study used a stratified two-stage probability sampling design as described earlier. As approximately the same number of participants was recruited from each selected location despite differences in the estimated population size of those locations, individuals did not have the same overall probability of selection across locations. Participants from smaller locations had a higher probability of being sampled than those from larger locations. Sampling weights were therefore applied to account for unequal probabilities of selection and to produce estimates that more appropriately represented the underlying FSW and MSM populations.

The weighting approach incorporated the overall sample size, the number of participants who responded, the sample size within each selected location, the estimated size of the key population, and the response rate within each selected location.

No post-stratification adjustment was applied because sufficiently detailed information on the relevant stratification characteristics was not available. In addition, the distribution of the study sample was broadly consistent with previous surveys conducted in the same geographic area with respect to key characteristics, including sub-county, location typology, and age. We therefore considered that post-stratification adjustment was unlikely to materially alter the resulting estimates.

The overall response rate exceeded 98% and was incorporated into the weighting procedure. Given this high response rate, any bias in the outcome estimates attributable to non-response is expected to be minimal.

### Ethical considerations

The study was approved by the Health Research Ethics Board at the University of Manitoba, Canada (HS25883), and the AMREF Ethics and Scientific Review Committee, Kenya (AMREF-ESRC P1365/2022). All participants provided informed consent before participation.

## Results

In total, 1,157 participants were enrolled in the study: 759 FSW (response rate 98.6%) across 64 PBS sessions and 398 MSM (response rate 94.7%) across 35 PBS sessions. Among PBS participants, 758 FSW (99.9%) and all 398 MSM (100%) completed the behavioural component of the BBS module; 758 FSW and 396 MSM completed the biological component. Of the 171 participants (101 FSW and 70 MSM) with reactive HIV rapid test results, 166 participants (98 FSW and 68 MSM) provided whole blood samples for further laboratory analyses. Urine tenofovir testing was conducted among 77 participants (58 FSW and 19 MSM) who reported current oral PrEP use in the BBS.

Table 1 presents participant characteristics from the BBS module. Most FSW participants were aged 25 years or older (82.2%), with a mean age of 32.3 years. MSM participants were younger, with a mean age of 25.5 years; 54.2% were younger than 25 years. Most participants primarily met sexual partners in physical locations (96.5% of FSW and 85.3% of MSM). However, 14.7% of MSM participants reported primarily using virtual platforms such as dating sites, social media or messaging applications to meet sexual partners.

**Table 1:** Characteristics of FSW and MSM participants, BBS module, Nairobi County, Kenya, 2023.

Participant characteristics		FSW *n* (%) *N* = 758	MSM *n* (%) *N* = 398
Sex work	Never had sex with a man in exchange for money or gifts	0.0 (0.0)	115 (29.0)
Age	<25 years	135 (17.8)	216 (54.2)
	25–34 years	345 (45.4)	151 (37.9)
	35+ years	279 (36.8)	31 (7.9)
	Mean age (SD)	32.3 (7.6)	25.5 (5.5)
Age at first sex with a man in exchange for money or gifts	<18 years	249 (32.8)	60 (15.0)
	18+ years	510 (67.2)	223 (56.0)
	Mean age at first sex in exchange for money or gifts	20.9	20.5
Predominant place for soliciting clients or meeting partners	Physical locations	732 (96.5)	339 (85.3)
	Virtual platforms	27 (3.5)	59 (14.7)
Duration of involvement in multiple sexual partnerships	<5 years	172 (22.6)	183 (46.1)
	5–9 years	200 (26.3)	140 (35.2)
	10 + years	388 (51.1)	74 (18.7)
Mean duration of involvement in multiple sexual partnerships		11.4	6.4
Number of sex acts per week	≤2	68 (8.9)	232 (58.4)
	3–4	175 (23.0)	105 (26.3)
	5+	516 (68.1)	61 (15.3)

All FSW participants reported exchanging sex with a man for money or gifts. However, nearly one-third (29.0%) of MSM reported never having exchanged sex with a man for money or gifts. One-third of FSW (32.8%) reported first exchanging sex with a man for money or gifts before the age of 18 years, compared with 15.0% of MSM. Among participants who reported having exchanged sex with a man for money or gifts, the mean age at first exchange was similar for FSW and MSM (20.9 and 20.5 years, respectively).

A higher proportion of FSW than MSM reported five or more sex acts per week (68.1% vs. 15.3%). More than half of FSW (51.1%) reported engaging in multiple sexual partnerships for 10 years or longer, compared with 18.7% of MSM.

### Behavioural outcomes

Table 2 summarises behavioural outcomes from the PBS module. Condom use at last sex with a paying client was reported by 80.8% of FSW. Among MSM, 68.9% reported condom use and 63.9% reported lubricant use at last sex with a paying client. Nearly half of FSW (47.9%) reported sex with a paying client without a condom in the previous 3 months, while 56.1% of MSM reported sex with a sexual partner without a condom during the same period. Limited availability of prevention commodities was a major barrier: 39.8% of FSW and 49.1% of MSM reported wanting to use a condom in the previous month but being unable to do so because condoms were not available at the time and place needed. Among MSM, 53.5% reported a similar gap for lubricants. Contact with a peer educator in the previous 3 months was reported by 68.8% of FSW and 65.8% of MSM.

**Table 2:** Behavioural outcomes among FSW and MSM participants, PBS module, Nairobi County, Kenya, 2023.

Behavioural outcomes	FSW *N* (%)	MSM *N* (%)
HIV-related knowledge		
Have the knowledge that having penetrative sex with a man without a condom increases the risk of acquiring HIV	757 (87.3)	398 (87.9)
Have the knowledge that consistent HIV treatment use by people living with HIV who are virally suppressed reduces the risk of HIV transmission	753 (76.5)	398 (77.4)
Condom use		
Used a condom at last sex with any paying client	756 (80.8)	239 (68.9)
Used lubricant at last sex with any paying client		238 (63.9)
Used a condom at last sex with a non-regular partner		397 (72.7)
Used lubricant at last sex with a non-regular partner		396 (61.4)
Used a condom at last sex with a regular partner		282 (62.0)
Used lubricant at last sex with a regular partner		287 (65.4)
Had sex with any paying client without using a condom in the previous 3 months	751 (47.9)	
Had sex with any sexual partner without using a condom in the previous 3 months		397 (56.1)
Wanted to use a condom with a sexual partner in the previous month but did not because a condom was unavailable at the time and place needed	754 (39.8)	398 (49.1)
Wanted to use lubricant with a sexual partner in the previous month but did not because lubricant was unavailable at the time and place needed		398 (53.5)
Contact with a peer educator		
Met with a programme peer educator in the previous 3 months	748 (68.8)	398 (65.8)

*N* represents the denominator and varies across questions because not all participants answered every question.

### Structural outcomes

Table 3 presents structural outcomes collected through the core PBS module. Stigma and discrimination from health service providers in the previous 3 months were reported by fewer than one in 10 participants (8.0% of FSW and 7.5% of MSM). By contrast, stigma and discrimination from family or community members were reported by approximately one-quarter of both groups (25.6% of FSW and 24.1% of MSM). Among those who experienced stigma or discrimination, 60.4% of FSW and 54.4% of MSM reported receiving support from the programme. Experiences of violence and arrest were also common. In the previous 3 months, 19.3% of FSW and 8.3% of MSM reported being arrested or physically assaulted by police or city askaris (authorities) while doing sex work or because they were MSM. Physically forced sex in the previous 3 months was reported by 10.7% of FSW and 6.5% of MSM. Among participants who experienced violence, 53.0% of FSW and 59.5% of MSM reported seeking and receiving support from the programme.

**Table 3:** Structural outcomes among FSW and MSM participants, PBS module, Nairobi County, Kenya, 2023.

Structural outcomes	FSW N (%)	MSM N (%)
Experience of stigma and discrimination and post-discrimination support		
Experienced discrimination from health service providers in the previous 3 months because of sex work or because they were MSM.	759 (8.0)	398 (7.5)
Experienced discrimination from family or community members in the previous 3 months because of sex work or because they were MSM	758 (25.6)	398 (24.1)
Received support from the programme after experiencing discrimination in the previous 3 months	310 (60.4)	151 (54.4)
Experience of violence and post-violence support		
Arrested or physically assaulted by police and/or city askaris in the previous 3 months while engaging in sex work or because they were MSM	759 (19.3)	398 (8.3)
Were physically forced to have sexual intercourse against their will in the previous 3 months	759 (10.7)	398 (6.5)
Received support from the programme after experiencing violence in the previous 3 months	340 (53.0)	121 (59.5)

*N* represents the denominator and varies across questions because not all participants answered every question.

### Biomedical outcomes

Table 4 presents self-reported biomedical outcomes from the PBS module. Approximately two-thirds of FSW (67.5%) and MSM (61.7%) reported visiting a key population programme clinic or drop-in centre (DIC) in the previous 3 months. Almost all participants reported ever having an HIV test (96.9% of FSW and 93.0% of MSM), but fewer reported testing in the previous 3 months (61.7% of FSW and 57.1% of MSM). Among participants who had not previously tested positive for HIV, oral PrEP use in the past 12 months was reported by 28.3% of FSW and 21.1% of MSM; among oral PrEP users, most reported consistent use for at least 3 months in the previous year. Self-reported emotional distress was frequently reported, with 73.0% of FSW and 53.0% of MSM reporting sadness, loneliness or stress almost every day during a two-week period in the previous 3 months. Among FSW, 19.3% reported an unintended pregnancy in the previous year, and 28.3% reported wanting contraception other than condoms in the previous 3 months but not receiving it at the place and time needed.

**Table 4:** Self-reported biomedical outcomes among FSW and MSM participants, PBS module, Nairobi, Kenya, 2023.

Biomedical outcomes	FSW *N* (%)	MSM *N* (%)
Clinic visit		
Visited or received services from the programme, clinic or drop-in centre in the previous 3 months	759 (67.5)	398 (61.7)
HIV testing		
Ever had an HIV test	759 (96.9)	398 (93.0)
Had an HIV test in the previous 3 months*	698 (61.7)	353 (57.1)
Linkage to ART		
Currently taking ART	111 (86.9)	76 (92.5)
Linkage to oral PrEP		
Taken oral PrEP in the previous 12 months	597 (28.3)	334 (21.1)
Taken oral PrEP consistently for at least 3 months in the previous year	101 (88.2)	47 (90.2)
Emotional distress		
Experienced sadness, loneliness or stress almost every day during a two-week period in the previous 3 months	758 (73.0)	398 (53.0)
Reproductive health		
Had an unintended pregnancy in the previous 12 months	754 (19.3)	
Wanted a contraceptive method (other than condoms) in the previous 3 months but did not obtain it at the place and time needed	756 (28.3)	

*N* represents the denominator and varies across questions because not all participants answered every question. *N excludes people living with HIV and those who reported the question as not applicable.

Table 5 presents laboratory-based biological outcomes from the BBS module. Nearly all participants who completed the biological component of the BBS module (753 FSW and 395 MSM) agreed to undergo HIV rapid testing. Reactive HIV test results were observed among 14.0% of FSW and 18.6% of MSM. Of the 171 participants with reactive rapid test results, 166 (97.1%) agreed to provide whole blood samples for viral load testing (98 FSW and 68 MSM). Valid viral load results were available for 99 (61.5%) participants. Of the 67 excluded samples, 34 (50.7%) were excluded due to insufficient specimen volume, 26 (38.8%) were excluded due to invalid laboratory results, and seven (10.4%) were excluded due to missing log forms/documentation. Among participants with reactive HIV rapid test results and valid viral load results, 83 (83.8%) were virally suppressed (85.9% of FSW and 80.4% of MSM). Key participant demographic and programme-related characteristics from valid and excluded whole blood specimen samples (*n* = 166) were compared using weighted data to account for the study’s sampling strategy (Supplementary Table 1). Compared with FSW participants, MSM participants were more likely to have a biological specimen excluded from analyses (*p* < 0.001). No statistically significant differences were observed between valid and excluded specimens when analysed by age, ART status, or recent clinic visits.

**Table 5:** Laboratory-based biological outcomes among FSW and MSM participants, BBS module, Nairobi, Kenya, 2023.

Biological indicators	FSW *n*/*N* (%)	MSM *n*/*N* (%)
HIV rapid test conducted	753 (99.3)	395 (99.7)
Reactive HIV rapid test results	101 (14.0)	70 (18.6)
Virally suppressed	60/70 (85.9)	23/29 (80.4)
Detectable urinary tenofovir	28/58 (48.3)	10/19 (52.6)

Among participants who reported current oral PrEP use (FSW *n* = 59, MSM *n* = 20; Table 4), 58 FSW and 19 MSM provided urine samples to test for the presence of tenofovir. Tenofovir was detectable in 48.3% of FSW and 52.6% of MSM samples despite high self-reported adherence (Table 5).

## Discussion

This study demonstrates the potential of expanded polling booth surveys (ePBS) to generate timely evidence on HIV programme performance by combining anonymous self-reported information with biological measures. The findings suggest important programme achievements, including high HIV prevention knowledge, high lifetime HIV testing and substantial contact with clinics serving key populations. At the same time, the study identified persistent gaps, including inconsistent condom and lubricant use, condom unavailability, low oral PrEP use, suboptimal viral suppression, police violence, and stigma from families and communities. Taken together, these findings indicate that FSW and MSM in Nairobi face substantial vulnerability to HIV acquisition and require sustained, differentiated and integrated prevention and treatment support.

The behavioural findings reinforce the need for continuous and reliable access to essential prevention commodities, particularly condoms and lubricants. Condom use at last sex with clients was higher among FSW than among MSM, but neither group reached the UNAIDS programme target for condom use at last paid sex or at last sex with a non-regular partner (23). Limited availability of prevention commodities appears to be an important contributing factor. Condom shortages have been repeatedly reported in Kenya and several other African countries, reducing availability and access (24). Around the time of this study, Kenya was experiencing an acute condom shortage that created substantial gaps in availability (25). In Mombasa, Kenya, Manguro et al. observed increased condom use among FSW and MSM participating in an oral PrEP intervention between 2017 and 2019, attributing the increase to extensive counselling on reducing the risk of HIV acquisition and widespread condom availability through the national programme (26). A systematic review of consistent condom uses among MSM in Africa found that higher self-worth and access to HIV counselling on reducing the risk of HIV acquisition were associated with more consistent condom use among MSM engaging in receptive anal sex (27). Among FSW in Kenya, consistent condom use also varies by partner type and is affected by factors such as alcohol use, number of sex acts and delays in payment by partners (28). Together, these findings suggest that condom and lubricant use is influenced not only by individual behaviour but also by broader programme and contextual factors, including commodity availability, partner dynamics and access to prevention support.

Violence and stigma emerged as major concerns affecting the health and wellbeing of both populations. In this study, violence and policing-related arrests were particularly high among FSW. Although fewer participants reported stigma from health service providers, stigma from family and community members was much more common. These patterns are consistent with evidence from Kenya and other settings suggesting that violence, police harassment and stigma reduce service access, weaken condom negotiation and increase HIV vulnerability among key populations (29–33). Criminalisation of sex work and same-sex relationships in Kenya has been identified as a root cause of violence and stigma affecting FSW and MSM (34–36). Only about half to three-fifths of participants who experienced stigma, discrimination or violence reported receiving support from the programme. This finding highlights an important gap in service delivery and suggests that responses to stigma, discrimination and violence should be monitored as core outcomes in key population programmes (37). Low levels of post-violence support may also indicate limited awareness of available services, low confidence in reporting mechanisms, gaps in crisis response systems, or fear of further stigma and retaliation (38).

Biomedical outcomes point to both programme coverage and missed opportunities for integration. A high proportion of FSW and MSM reported ever testing for HIV, but far fewer reported testing in the previous 3 months, despite national recommendations for quarterly testing (6). Consistent with our findings, other studies in Kenya have shown high lifetime HIV testing among FSW and MSM but lower levels of frequent testing (11, 39, 40). Although testing frequency may have been affected by broader HIV test kit shortages (41, 42), this finding still highlights the need for more differentiated and accessible testing models for FSW and MSM. Self-reported emotional distress was commonly reported, especially among FSW, consistent with evidence from Kenya linking violence, stigma, substance use and sexual behaviours associated with HIV exposure to poor mental health among key populations (43). A recent scoping review synthesising evidence from Africa suggests that integrated mental health and HIV care can improve mental health outcomes and adherence to antiretroviral therapy (44). For FSW and MSM, these findings support incorporating feasible mental health screening and first-line psychosocial support within routine HIV services. Among FSW, unintended pregnancy and unmet need for contraception suggest that integration of HIV programmes with sexual and reproductive health services remains inadequate (45, 46). Evidence from Kenya shows that offering non-barrier family planning methods within HIV programmes can help reduce unintended pregnancies among FSW (47). Programmes should therefore move beyond narrowly defined HIV service delivery and offer integrated, gender-responsive services that address mental health, contraception, HIV prevention and treatment together.

Laboratory outcomes highlight gaps in treatment and oral PrEP use. Viral suppression among participants living with HIV remained below the UNAIDS 95% target. Other studies from Kenya have reported that harmful or dependent alcohol use is associated with lower ART adherence and viral suppression among MSM (48), and that provider and peer support are important for sustaining engagement in care among MSM living with HIV (49). Oral PrEP uptake remained low despite high availability (8), and only about half of participants who self-reported current oral PrEP use had detectable tenofovir in urine. Self-reported adherence can be inflated due to social desirability or recall bias (50), with objective metrics of adherence like the urine tenofovir assay being employed to measure adherence more accurately (51). This finding suggests challenges with oral PrEP uptake and adherence in our study and highlights the importance of monitoring effective oral PrEP use. Shah et al. also found low oral PrEP uptake among FSW in Nairobi and recommended addressing social and structural barriers to oral PrEP utilisation (52). As Kenya introduces newer prevention options, including injectable PrEP, routine outcome monitoring should assess not only uptake but also continuation, effective use, equity of access and whether new products are reaching those who face the greatest barriers to existing methods.

A key contribution of ePBS is its ability to generate actionable programme intelligence rather than solely epidemiological estimates. Importantly, the value of the approach lies not only in the data it generates, but also in how those data are used. By combining behavioural, structural and biological outcomes, ePBS supports a programme science approach (53) in which evidence is used to continuously identify gaps, prioritise responses, and adapt service delivery. This is especially important in constrained funding environments, where programmes must make difficult choices about which services to sustain, adapt or integrate. A distinguishing feature of ePBS is the meaningful involvement of community members throughout the knowledge generation and utilisation cycle. Community members helped develop the tools, participated in data collection, interpreted findings through the lens of lived experience and helped identify practical actions for programme improvement. The Global Fund has recognised polling booth surveys and the expanded version as a method for measuring programme outcomes among key and priority populations (14), and the approach has also been adapted for adolescent girls and young women and general population programmes (54, 55). In Nairobi, ePBS findings informed microplanning, commodity distribution, differentiated service delivery, violence response, PrEP programme implementation, and integration of mental health and sexual and reproductive health services within existing programmes. Sharing findings with peer educators, clinic teams and county officials created opportunities for joint reflection and helped build shared ownership of subsequent actions. This process shifts monitoring beyond performance reporting and creates opportunities to improve services while they are being delivered. Used iteratively, ePBS can complement routine programme data by measuring outcomes that are not consistently captured in routine monitoring systems.

This study has several limitations. First, its cross-sectional design means that the findings are descriptive and cannot establish causal relationships between programme exposure and outcomes. Second, although the study used multiple methods, including PBS and BBS, to reduce social desirability bias and encourage honest reporting, it still relied heavily on self-reported data for sensitive behaviours, service use, stigma and violence. Under-reporting or over-reporting may therefore have occurred. A substantial proportion of whole blood samples were unavailable for analysis because of insufficient specimen volume or invalid laboratory results. Of the 158 participants who provided whole blood samples, only 99 (62.7%) yielded valid viral load results. This reduced the precision of viral load suppression estimates and may have introduced selection bias. Specimen loss appeared to arise primarily from operational challenges during venous blood collection, transport, and laboratory processing. Future applications of ePBS should strengthen training around specimen collection processes and laboratory quality assurance procedures. Sample selection for this study focused primarily on physical solicitation and meeting locations. As a result, FSW and MSM who primarily seek sexual partners through virtual platforms or other hidden networks may have been underrepresented or excluded. The findings may therefore underrepresent the experiences of individuals who primarily use virtual platforms or other less visible networks. Previous research among MSM in three Kenyan cities found that 14.7% of participants reported using only physical sites to meet sexual partners, 64.0% used both physical and virtual sites and 21.2% used only virtual sites (56). These findings also highlight the importance of including virtual sites into future studies involving MSM and FSW to improve representativeness and reduce the risk of under-sampling individuals who primarily engage through online or other hidden networks. Another limitation is that the study grouped MSM and male sex workers (MSW) together in the analysis, consistent with the Kenyan programmatic definition of MSM. However, this may have masked important heterogeneity within the population. Future studies should consider collecting and analysing data on sex work separately within the broader MSM population where sample size permits. Finally, the mental health question was not derived from a validated screening tool but was included to capture a brief, programme-relevant indication of emotional wellbeing within the constraints of the PBS format. It was intended for descriptive purposes only and should not be interpreted as a diagnostic measure of any specific mental health condition.

## Conclusion

FSW and MSM in Nairobi remain priority populations for HIV prevention and treatment. The findings highlight both achievements and areas requiring further attention within HIV programmes serving FSW and MSM in Nairobi. Addressing these gaps will require adaptive, differentiated and community-led service delivery approaches, alongside integrated programming that combines biomedical services with structural interventions. For example, condom unavailability remains an important and potentially modifiable programmatic barrier, underscoring the need to strengthen reliable access to condoms at the times and places they are needed. Similarly, identifying low oral PrEP adherence using a urine-based assay can prompt counselling to strengthen adherence (22). Routine monitoring of key population programmes is critical for tracking progress, identifying programme gaps and adapting services to the needs of key populations to achieve programme targets. The ePBS approach offers a practical way to generate timely and actionable evidence while maintaining meaningful community involvement in the interpretation and use of findings. When used routinely, ePBS can help programmes identify emerging challenges, refine service delivery, and strengthen accountability to the communities they serve.

## Data Availability

The raw data supporting the conclusions of this article will be made available by the authors, without undue reservation.
