# Supplementary material for: Expanded polling booth surveys monitor HIV programme outcomes among female sex workers and men who have sex with men in Nairobi, Kenya
Source: Front Public Health. 2026 Oct 27;14:1969489. doi: 10.3389/fpubh.2026.1969489 (PMC13748319; doi:10.3389/fpubh.2026.1969489)
Supplement: Supplementary file 1 [file Data_Sheet_1.PDF]

Supplementary Table 1

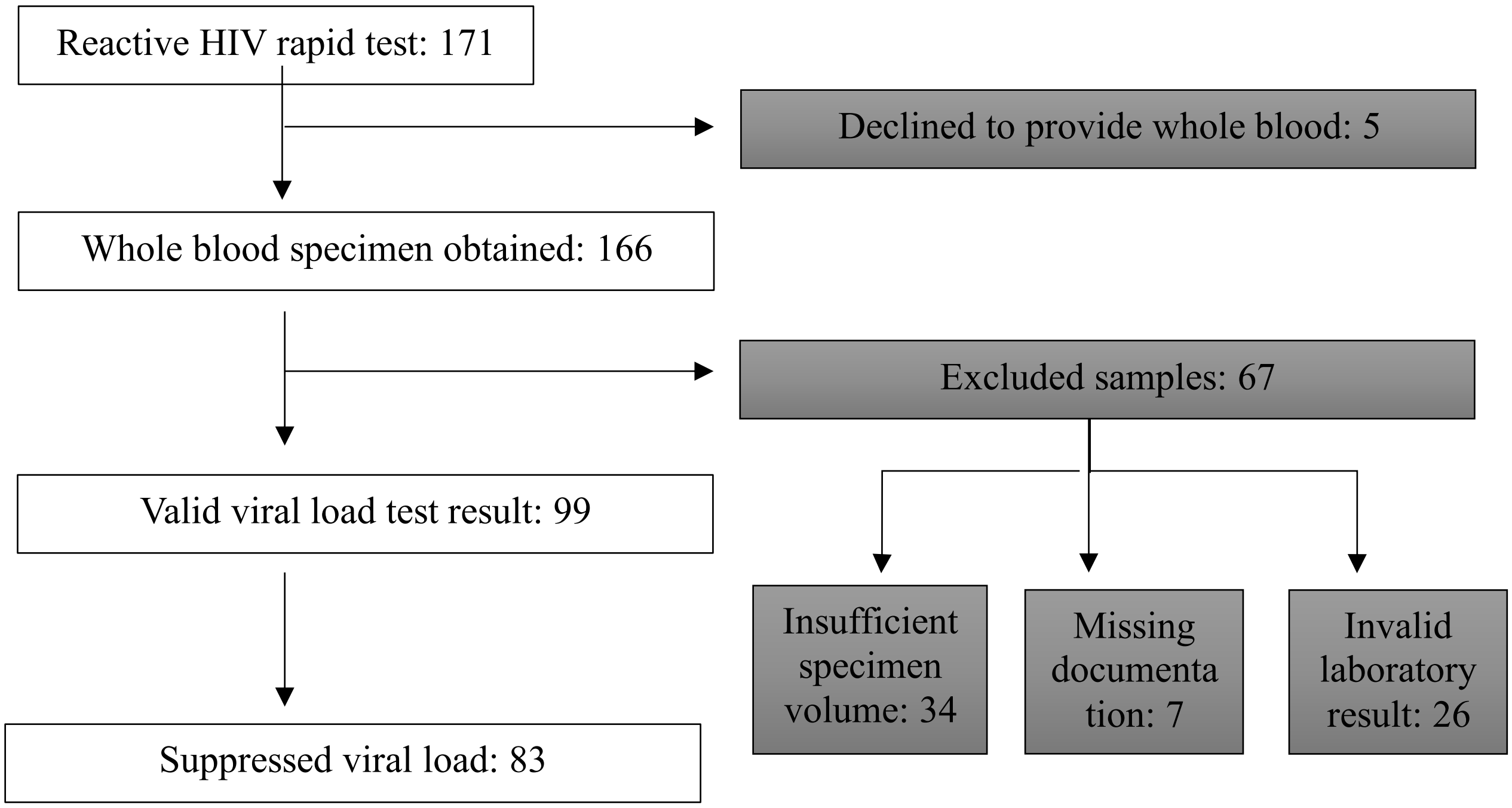

	**Valid viral load** ***n=99*** **(%)**	**Excluded specimen** ***n=67*** **(%)**	**p-value**
FSW (n=98)	69.0	31.0	<0.001
MSM (n=68)	40.6	59.4	
**FSW (n=98)**			
Age (mean)	37.9	36.7	0.58
Currently on ART	94.9	100	0.21
Recent clinic visit	61.5	44.4	0.77
**MSM (68)**			
Age (mean)	28.6	29.4	0.56
Currently on ART	95.8	97.4	0.72
Recent clinic visit	38.5	55.6	0.31
